# Effectiveness of Physical Exercise Interventions on Pulmonary Function and Physical Fitness in Children and Adults with Cystic Fibrosis: A Systematic Review with Meta-Analysis

**DOI:** 10.3390/healthcare10112205

**Published:** 2022-11-03

**Authors:** Guillermo García-Pérez-de-Sevilla, Thomas Yvert, Ángela Blanco, Alicia Irene Sosa Pedreschi, Israel J. Thuissard, Margarita Pérez-Ruiz

**Affiliations:** 1 Faculty of Sports Sciences, Department of Physiotherapy, Universidad Europea de Madrid, 28670 Madrid, Spain; 2 Departamento de Salud y Rendimiento, Facultad de Ciencias de la Actividad Física y del Deporte–Instituto Nacional de Educación Física (INEF), Universidad Politécnica de Madrid, 28040 Madrid, Spain; 3 Faculty of Sports Sciences, Department of Research, Universidad Europea de Madrid, 28670 Madrid, Spain; 4 Faculty of Biomedical Sciences, Universidad Europea de Madrid, 28670 Madrid, Spain

**Keywords:** cystic fibrosis, exercise, pulmonary function, cardiorespiratory function, muscle strength, physical fitness

## Abstract

**Objective:** Physical exercise is associated with several benefits in the treatment of cystic fibrosis (CF), associated with a reduction in patient mortality. The aim of this systematic review was to determine the effectiveness of exercise interventions on physical condition and lung function in children and adults with CF to establish the most appropriate type and dose of physical exercise used so far. **Methods:** The studies included were randomized controlled trials with physical exercise interventions performed with children or adults with CF, analyzing the effects on pulmonary function, cardiorespiratory capacity, and muscle strength. The variables analyzed in at least four studies in the same population (children or adults) with the same measuring test were included in the meta-analysis. **Results:** *Pulmonary function:* There were no changes in the forced expiratory volume 1 s, but mouth expiratory/inspiratory pressures were improved in some studies. *Physical fitness:* In children, the interventions did not manage to improve the VO_2peak_ (SMD = 0.22; 95%CI: −0.25 to 0.68; *p* = 0.73) but improved muscle strength. In adults, physical exercise interventions based on high-intensity aerobic training showed positive results in the VO_2peak_, and in some muscle strength outcomes. **Conclusions:** Exercise interventions in children and adults with CF are effective in improving muscle strength, cardiovascular capacity, and respiratory muscle function. However, they do not achieve improvements in lung function. The most effective programs are those using strength training or cardiovascular high-intensity interval training, although to date there have been few such interventions.

## 1. Introduction

Cystic fibrosis (CF) is an autosomal recessive disease caused by mutations of the *Cystic Fibrosis Transmembrane Conductance Regulator* (*CFTR*) gene, located on chromosome 7 [1]. The chloride channel defect caused by these mutations affects all epithelial cells, disrupting ion transport in various tissues and causing blockage of secretory glands [2]. Although CF is a multi-organ disease, the lung involvement determines the morbidity and mortality of the disease [3].

When CF was described in 1938 by pathologist Dorothy Hansine Andersen, it was a globally fatal disease of early childhood [4]. Thanks to medical progress, patients have increased survival, so that death in childhood has become very rare [5]. At present, treatment with CFTR modulators is rapidly changing the outlook for people with CF. However, taking a combination of drugs for life is not an ideal approach, as the cost is enormous, and some patients may not tolerate the treatment in the long term. Therefore, other corrective strategies, such as physical exercise, continue to be explored [6].

Physical exercise is associated with several potential benefits in the treatment of CF, such as positive effects on lung function [7], mucus clearance [8], maintenance of bone health [9], healthier body composition [10], decreased systemic inflammation [11], and improved physical fitness [12], all of which are associated with a reduction in patient mortality [13]. Therefore, physical exercise is currently proposed as a very useful non-pharmacological tool to keep patients in high physical condition, allowing them to access new therapies in the best physical condition. However, physical exercise interventions carried out in CF patients are very heterogeneous and there is limited evidence about their effectiveness on VO_2max_ and lung function [14]. In this sense, an adequate physical condition, measured by VO_2max_, is a positive prognostic factor for the evolution of the disease, associated with a reduction in the number of annual hospitalizations [15]. The dose and type of physical exercise used is critical to achieving positive physiological adaptations. In CF patients, most protocols implement moderate-intensity aerobic or strength exercise [16], performed at home, without supervision [7]. It is noteworthy that perhaps aerobic exercise in these patients should be performed in the form of short, high-intensity interval exercise, to achieve greater physiological adaptations, as is being pioneered for patients with chronic obstructive pulmonary disease [17]. Although exercise is part of the routine care of CF patients, more precision in exercise dosage is needed today.

The current literature contains many studies on exercise interventions in CF children, but few interventions have been carried out in adults. This is possibly because disease progression and pulmonary deterioration do not allow patients to adhere to the exercise programs currently prescribed by hospitals. Also, until very recently, the CF population was not reaching adulthood [18]. For these reasons, to date there are few interventions that show effectiveness on prognostic variables of the disease in the adult population.

Therefore, the aim of the present study was to determine the effectiveness of exercise interventions on physical condition and lung function in children and adults with CF, updating the scientific evidence about this topic [14].

## 2. Material and Methods

This systematic review was carried out according to the Preferred Reporting Items for Systematic Reviews and Meta-analyses (PRISMA) statement [19] and was registered in PROSPERO (International prospective register of systematic reviews) on 10 June 2022, with the registration number CRD42022336479.

### 2.1. Data Source

Studies in four databases were reviewed: PubMed, Scopus, SPORTDiscus, and Web of Science, published from 2017 to 19 July 2022. The search terms were: “cystic fibrosis AND (exercise OR “physical activity” OR sports) AND (randomized OR “clinical trial”)”. Reviews and grey literature were excluded. After removal of the duplicates, two authors (GGPS and ABV) independently screened the titles and abstracts and then evaluated the full text of potentially relevant studies. Disagreements were resolved by consultation with a third reviewer (MPR). Studies were eligible for inclusion if they met the inclusion criteria and did not meet the exclusion criteria. All the excluded studies were recorded, with the reasons for exclusion.

### 2.2. Inclusion and Exclusion Criteria

The criteria for inclusion were (1) population—children or adults with CF; (2) type of study—randomized controlled trials (RCT) only; (3) type of intervention—physical exercise program with real exercise practice; (4) outcomes—short term or long-term measurement of pulmonary function, cardiorespiratory capacity, or muscle strength.

Finally, the exclusion criteria were: (1) abstract not in English; (2) exercise protocol not included in the paper; (3) studies involving CFTR modulator therapies; (4) studies published before 2017.

### 2.3. Data Extraction

The full text of all the studies matching the inclusion criteria was retrieved, and then stored electronically and systematically reviewed.

Pre- and post-exercise mean and standard deviation of descriptive characteristics of the participants and the following outcomes were extracted and recorded, by two authors (GGPS and ABV):

#### 2.3.1. Pulmonary Function

FEV_1_, forced vital capacity, maximal expiratory and inspiratory mouth pressures (MEP and MIP), and respiratory muscle endurance test.

#### 2.3.2. Physical Fitness

*Cardiorespiratory outcomes*: 6-Minute-Walking Test (6MWT) distance, VO_2peak_, modified shuttle run distance, and endurance exercise capacity.

*Muscle strength and functionality outcomes*: horizontal jump test, medicine ball throw, handgrip strength, bench press strength, pectoral strength, dorsal strength, time up and go test, leg press, sit ups, pushups, biceps curls, and quadriceps strength.

The characteristics of the physical exercise programs were also extracted and recorded.

Three authors were contacted by e-mail to request missing data. Two of them provided the necessary data.

### 2.4. Data Synthesis

Characteristics of studies, participants, and interventions were summarized in tables. The Revised Cochrane risk-of-bias tool for randomized trials (RoB 2) [20] was used to assess the risk of bias. This assessment was performed separately by two investigators (GGPS and ABV). In cases of disagreement, a third investigator (MPR) assessed the study, and the disagreement was resolved by consensus.

### 2.5. Statistical Analysis

The meta-analysis of this review has been limited to the outcomes analyzed in at least four studies, in the same population (children or adults), with the same measuring test, which was only the VO_2peak_ in children with CF.

Meta-analyses were conducted using the statistical software package Stata 16.1 (StataCorp, College Station, TX, USA).

The mean of differences and pooled standard deviation (SD) was calculated using pre- and post-intervention data.

At first, effect sizes (ESs) and adjusted Hedges’ g (95% confidence intervals (CIs)) were calculated for each study by means of t-scores, number of subjects, and standard deviation (SD).

Then, the Q and I^2^ statistics were calculated to find the proportion of the observed variability due to heterogeneity but not to randomness. I^2^ values of <25%, 25–50%, and >50% are considered to represent small, medium, and large inconsistency, respectively [21].

In addition, an analysis of heterogeneity was carried out by interpreting the L’Abbé and Galbraith graphs [22].

Finally, publication biases were identified using the funnel plot test and the Egger test, allowing us to detect asymmetries in the funnel graph [23]. Significance level was set at 5% (0.05).

## 3. Results

### 3.1. Flow of Studies through the Review

**Figure 1:**
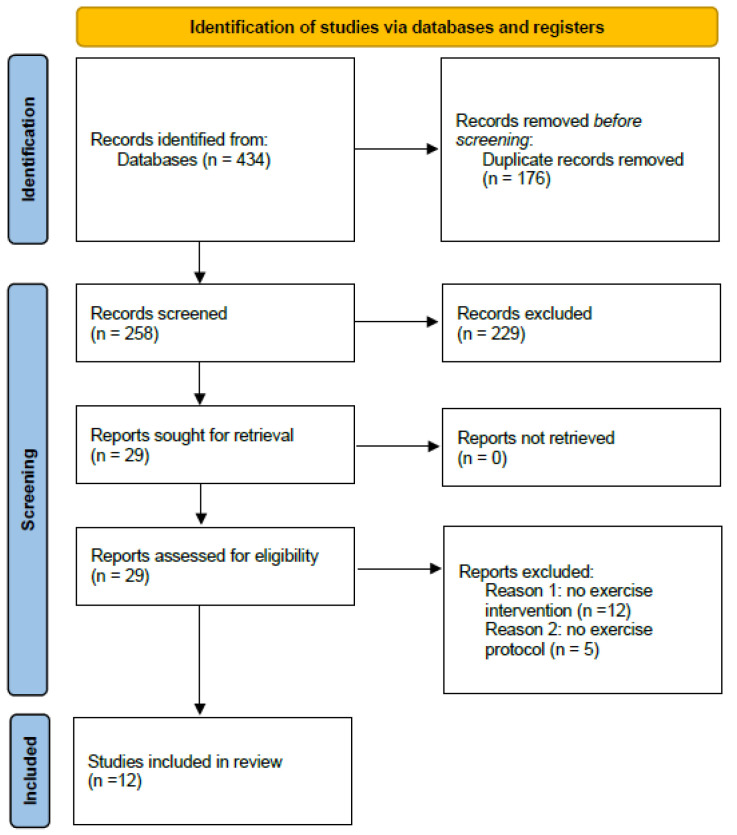
Flow diagram of the studies included.

The search strategy identified 434 articles from the databases. After removal of duplicates, 258 articles were initially screened via title and abstract, and 29 were iden-tified as potentially relevant. Full-text examination further excluded 17 studies, leaving 12 studies for inclusion in this analysis, all RCTs (Figure 1).

### 3.2. Characteristics of the Included Studies

#### 3.2.1. Subjects

Participant demographics, intervention characteristics, and outcomes are outlined in Table 1 (children with CF) and Table 2 (adults with CF).

Pulmonary, cardiorespiratory, and strength outcomes were analyzed in seven studies [24,25,26,27,28,29,30] with a total of 219 children aged 11–14 years (51% male), and in five studies [31,32,33,34,35] with a total of 180 adults aged 22–57 years (45% male).

#### 3.2.2. Interventions

Interventions in children with CF were conducted for 2 to 12 months, and the modes of exercise were resistance training (four studies [25,26,27,30]), resistance training + neuromuscular electrical stimulation (one study [27]), and respiratory muscles training (three studies [24,28,29]). The participants trained for 60–210 min per week at a low to moderate intensity, divided into 3 to 5 sessions per week.

Interventions in adults with CF were conducted for 2 to 6 months, and the modes of exercise were aerobic training (three studies [32,33,34]), and combined resistance + aerobic training (two studies [31,35]).

The participants trained for 30–180 min per week divided into 2 to 3 sessions per week, at a moderate (four studies [31,32,34,35]) to high (one study [33]) intensity.

#### 3.2.3. Outcome Measures

The variables were grouped into two different categories, according to the objective of the study:

- Pulmonary function:

Forced-expiratory volume 1 s (FEV_1_), maximal expiratory and inspiratory mouth pressures (MEP and MIP), forced vital capacity, and respiratory muscle endurance.

- Physical fitness:

*Cardiorespiratory capacity:* VO_2peak_, 6MWT distance, endurance exercise capacity, and the modified shuttle run distance.

*Muscle strength and functionality:* leg press, bench press, pectoral strength, handgrip strength, push-ups, sit ups, biceps curls, quadriceps strength, dorsal strength, time up and go, medicine ball throw, and horizontal jump test.

### 3.3. Quality Assessment of Study Methodology

**Figure 2:**
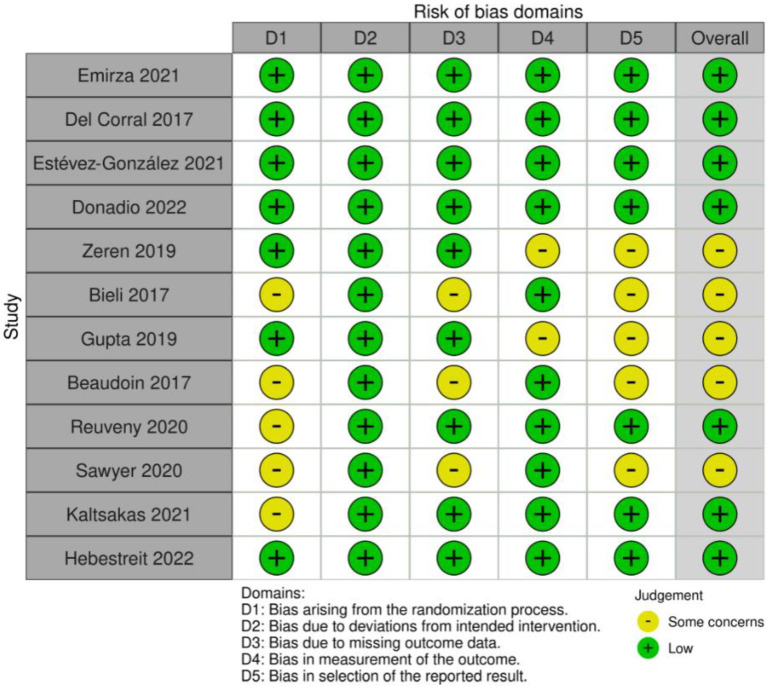
Risk of bias assessment of the included studies.

**Figure 3:**
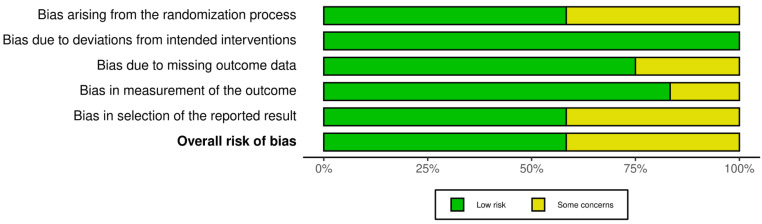
Risk of bias summary.

The risk of bias analysis revealed that seven studies (58%) [24,25,26,27,32,34,35] presented low risk, and five studies (42%) [28,29,30,31,33] were classified as presenting some concerns. The most common biases were: (i) “bias arising from the randomization process” where five studies (42%) [29,31,32,33,34] were classified as “some concerns”, mainly because the process was not sufficiently described; (ii) and “bias in selection of the reported result”, where five studies (42%) [28,29,30,31,33] were classified as “some concerns” (Figure 2 and Figure 3).

### 3.4. Effect of the Intervention

#### 3.4.1. Pulmonary Function

In children with CF, four studies [24,28,29,30] analyzed the FEV1 and the forced vital capacity, without changes. Meta-analysis of this outcome was not performed because of missing standard deviations. One study [28] observed a significant increase in MIP (+37.63 ± 8.21 cm H_2_O; *p* < 0.001), while another study [24] observed no changes. Concerning MEP, one study [24] observed a significant increase in MEP (+12.42 ± 9.52 cm H_2_O; *p* = 0.003), while another study [28] observed no changes. Finally, one study showed a significant improvement of respiratory muscle endurance [29] (+7.03 ± 8.15 min; *p* < 0.01) (Table 1).

In adults with CF, one study [35] analyzed the FEV_1_, without observing any improvement. In contrast, another study [34] showed positive results in the MIP and the MEP.

#### 3.4.2. Physical Fitness

Cardiorespiratory Capacity

**Figure 4:**
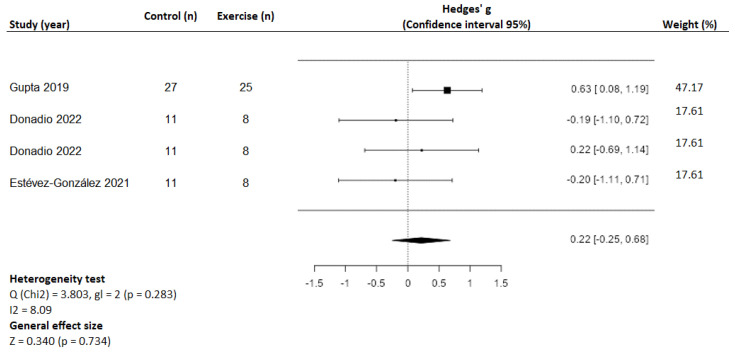
Meta-analysis and forest plot of VO_2peak_ in children with cystic fibrosis [26,27,30].

In children with CF, one study [25] observed significant improvements in 6MWT (+40.4 m; 95%CI: 21.42 to 59.38; *p* > 0.01), while two studies [24,28] observed no changes. Concerning the modified shuttle run distance, one study [25] observed no changes. Finally, three studies [26,27,30] analyzed the VO_2peak_, and only one study [30] showed significant improvements in this outcome (+4.15 mL/kg/min; 95%CI: 1.22 to 7.08; *p* = 0.006) (Table 1). The results of our meta-analysis show no significant changes in the VO_2peak_, although the effect size was moderate (SMD = 0.22; 95%CI: −0.25 to 0.68; *p* = 0.73), with low heterogeneity (Q = 3.80; *p* = 0.283; I2 = 8%) (Figure 4).

In adults with CF, four studies [32,33,34,35] analyzed the VO_2peak_, with two studies [34,35] observing positive results. Concerning endurance exercise capacity, one study [35] showed an improvement, while another study [33] showed maintenance. Finally, one study [34] showed a significant increase in the 6MWT.

Muscle Strength and Functionality

In children with CF, one study [25] showed significant improvements in the horizontal jump test (+9.22 cm; 95%CI: 1.95 to 16.5; *p* < 0.05), but no changes in the medicine ball throw. Two studies [25,27] observed significant improvements in handgrip strength (+6.83 kg; 95%CI: 4.18 to 9.48; *p* < 0.01) (+12.1 ± 13.6 kg; *p* = 0.03) and two studies [26,27] observed significant improvements in the pectoral strength, dorsal strength, and bench press. Finally, one study [27] observed an improvement in the time up and go test (Table 1).

In adults with CF, one study based [31] on resistance training observed significant improvements in the leg press (+33.3 kg; *p* = 0.02), bench press (+6.8 kg; *p* < 0.05), and number of push-ups, but no changes in the handgrip strength, biceps curls, and number of sit-ups. Another study based [34] on aerobic training showed an improvement in quadriceps strength.

## 4. Discussion

The aim of this systematic review was to analyze the effectiveness and characteristics of physical exercise interventions on pulmonary function and physical condition (cardiorespiratory function and muscle strength) carried out in children and adults with CF over the last 5 years. All the studies included in this review were RCTs of fair–good methodological quality. They showed that patients enrolled in exercise programs obtained better improvements in physical condition compared to patients undergoing conventional therapy (only including recommendations of physical activity). In addition, we have noted that few studies have analyzed both lung function and physical fitness as target variables. Regarding the characteristics of the exercise interventions proposed to date, they are mixed programs combining cardiorespiratory and strength exercise with a predominance of moderate intensity. The results of this review allow us to be aware of the components of the workload (type, frequency, intensity, and duration of exercise) used to date in children and adults with CF. We believe these results can be of great support to progress in this field and to be able to propose new, and perhaps more effective, approaches.

Both in children and adults with CF, the physical exercise interventions included in this systematic review failed to improve lung function measured by FEV_1_ [24,28,29,35]. However, some studies found significant improvements in respiratory muscle function [24,28,29]. In adults, one study found improved MIP (+13 cm H_2_O) and MEP (+30 cm H_2_O) using interval training at an intensity of 70% of VO_2peak_ for 12 weeks [34]. In children, one study observed an improvement in MIP (+37.63 ± 8.21 cm H_2_O) after inspiratory muscle training with a *Threshold Inspiratory Muscle Trainer* at an intensity of 30% of MIP, 15 min, two times a day for eight weeks [28]. Another study performed in children found that MEP improved (+12.42 ± 9.52 cm H_2_O) after training the expiratory muscles with a *Threshold Respiratory Muscle Trainer* at an intensity of 30% of MEP, 20 min, five times a week for six weeks [24]. Finally, another intervention of respiratory muscle training using a device, for 10 min, twice a day, for two weeks, showed an improvement in respiratory muscle endurance (+7.03 ± 8.15 min) [29]. These last three studies, based on specific training of the respiratory muscles in children [24,28,29], despite improving respiratory muscle function, did not observe improvements in FEV_1_, forced vital capacity, nor in the other pulmonary function tests. The reason that these studies are successful in improving respiratory muscle function but not lung function per se may be since most of these studies are conducted in patients with mild-to-moderate CF [9]. However, it is important to note that improved respiratory muscle function is associated with increased cardiorespiratory efficiency and decreased dyspnea in populations affected by chronic obstructive pulmonary disease (COPD) [36].

Concerning physical condition and particularly cardiorespiratory function, it is worth noting that physical capacity is one of the main predictors of risk of exacerbation and mortality in CF patients [13,14]. Three strength training-based interventions [26,27,30] analyzed VO_2peak_ in children with CF, of which two studies [26,27] showed no improvement after 8 weeks of treatment, despite improving muscle strength variables. The third intervention [30] was the only one reporting significant improvements in VO_2peak_ (+2.71 mL/kg/min) and was based on strength exercise recommendations performed at home, three days per week for 12 months, without providing data on the duration or intensity of the sessions. Nevertheless, this intervention did not show improvements in lung function.

The results of our meta-analysis (see Figure 4) on this variable in children showed no significant changes, perhaps due to an insufficient number of studies or to lower sample sizes, although a moderate effect size is noteworthy (SMD = 0.22; 95%CI: −0.25 to 0.68; *p* = 0.73).

In adults, two studies conducting eight-week cardiovascular exercise-based interventions at moderate intensity showed no significant improvement in VO_2peak_ [32,33]. However, the studies carried out with adults with CF by Kaltsakas et al. [34] and Hebestreit et al. [35], based on moderate-vigorous intensity cardiovascular training, 90–180 min per week for 6 and 12 months, found significant improvements in VO_2peak_ (+2.8 mL/kg/min, *p* < 0.05 in Kaltsakas et al. [34]), as well as increased performance in functional physical tests.

Another variable of interest is muscle strength. Improved muscle strength in CF patients indicates peripheral physiological adaptations in a tissue markedly affected in CF, for example in its mitochondrial function [37]. In children, three studies [25,26,27] based on supervised moderate-to-high-intensity strength training, with frequencies from three to five days per week for eight weeks, obtained significant improvements in different muscle strength variables (horizontal jump, hand grip strength, bench press, pectoral strength, dorsal strength, Time up and Go test). One of these studies [25], whose strength training was performed in a circuit at an intensity of 70–80% of maximum heart rate, also achieved a clinically significant improvement [38] in the 6MWT performance (+40.4 m; 95%CI: 21.42 to 59.38; *p* > 0.01). Possibly, strength exercise can be well tolerated in patients with CF, and if performed prior to cardiovascular training may be useful to strengthen peripheral muscles and reduce ventilatory demands during exercise, and thereby improve cardiovascular capacity [39]. In the only study comparing the combination of strength training with electrostimulation versus strength exercise alone, no significant differences were observed between the two groups of children with CF [27]. In adults, only one study [31] analyzed muscle function in detail, with a moderate-intensity mixed strength and cardiovascular physical exercise intervention consisting of three sessions per week for 12 weeks, achieving significant gains in upper and lower limb strength. Based on the data analyzed in this review, although optimal training protocols for CF patients have not yet been determined, it is likely that a combination of high-intensity interval training (HIIT) and strength training would be most effective in achieving health benefits and long-term exercise adherence [39].

This systematic review provides novel findings regarding physical exercise interventions in CF patients. However, it suffers from some limitations. Mainly, we did not find enough RCTs that analyzed the same variables with the same instruments to perform quantitative analysis, in both children and adults. Furthermore, most of the studies had low sample sizes. Similarly, very few studies have analyzed pulmonary, cardiorespiratory, and muscular function at the same time. This would be very useful to observe interactions between these variables, and to determine which adaptations to exercise occur with which types of programs and which do not. Similarly, there was marked heterogeneity in the interventions in terms of type and dose of exercise, as well as in terms of exercise supervision, with unsupervised programs presenting risks of bias. Finally, we believe shorter exercise interventions are needed, with intensities above 70% of VO_2peak_ and prolonged recoveries, as is being done in other pulmonary pathologies [17]. Continued work at moderate doses is sometimes not well tolerated by the pulmonary patients as it causes high ventilatory demand [34]. Interval training may be better tolerated and may be superior in terms of physiological adaptations, thereby improving performance of daily activities in people with CF [40].

The results of this systematic review indicate that current physical exercise interventions in CF patients improve muscle strength and respiratory muscle function in both children and adults, and cardiorespiratory function in adults, although they do not improve lung function. Therefore, physical exercise achieves positive physiological adaptations at the peripheral level in these patients, possibly involving muscle oxidative enhancement and stimulation of mitochondrial function. Future studies should use interventions that combine strength exercise with HIIT, with the aim of increasing the exercise dose without generating a high pulmonary demand. This may lead to greater improvement in cardiovascular and pulmonary function. Similarly, efforts should be made to improve adherence to exercise to ensure it becomes part of the patient’s lifestyle, since those studies lasting more than 12 months achieve greater changes in important variables such as VO_2peak_. On the other hand, it is also important to undertake trials with different types and doses of exercise in adults with CF, since, as we have seen in this review, these studies are still scarce, and less relevant improvements are observed compared to children, due to the deterioration that already exists in the lung.

## 5. Conclusions

Exercise interventions in children and adults with CF are effective in improving muscle strength and respiratory muscle function. In adults with CF, they also improve cardiovascular capacity. However, these interventions do not achieve improvements in lung function. The latter could be since most of the patients had mild-moderate lung involvement and might need very specific exercise doses to achieve significant improvements. The most effective programs are those using strength training or cardiovascular high-intensity interval training, although to date there have been few such interventions.

## Figures and Tables

**Table 1:** Outcomes from physical exercise interventions on children with cystic fibrosis.

Authors, Year	Population	Intervention	Comparison	Outcomes
**Emirza et al.** **2021** [24]	IG: n = 14 CG: n = 14 12 years 57% female	Duration: 6 weeks Frequency: 5 days/week Intensity: 30% of MEP Volume: 20 min Type: Respiratory muscle exercises with a device Supervised: no	Sham respiratory muscle exercises	MIP ↔ MEP ↑ FEV1 ↔ Forced vital capacity ↔ 6MWT ↔
**Del Corral** **t al. 2017** [25]	IG: n = 17 CG: n = 18 11.5 years 53% male	Duration: 6 weeks Frequency: 5 days/week Intensity: 70–80% HRmax Volume: 30–60 min Type: Video game exercise program, performing running, squats, lunges, and biceps curls Supervised: yes, virtual	Habitual physical activity recommendations for cystic fibrosis	6MWT ↑ Modified Shuttle Run Distance ↔ Horizontal jump test ↑ Medicinal ball throw ↔ Handgrip strength ↑
**Estévez-González et al. 2021** [26]	IG: n = 8 CG: n = 11 12 years 68% male	Duration: 8 weeks Frequency: 3 days/week Intensity: 60–80% 1RM Volume: 3 sets of 8–12 reps of 5 exercises Type: Resistance training of major muscle groupsSupervised: Yes	Habitual physical activity recommendations for cystic fibrosis	VO_2peak_ ↔ Bench Press ↑ Pectoral strength ↑ Dorsal strength ↑
**Donadio et al. 2022** [27]	IG1: n = 8 IG2: n = 8 CG: n = 11 12.6 years 70% male	IG1: Duration: 8 weeks Frequency: 3 days/week Intensity: 40–80% 1RM Volume: 2–3 sets of 12–15 reps of 6 exercisesType: Resistance training of major muscle groups Supervised: Yes IG2: same protocol + NMES applied to quadriceps, latissimus dorsi and trapezius	Habitual physical activity recommendations for cystic fibrosis	IG1 and IG2: VO_2peak_ ↔ Time up and Go ↑ Bench Press ↑ Pectoral strength ↑ Dorsal strength ↑ Handgrip strength ↑ No differences between IG1 and IG2
**Zeren et al.** **2019** [28]	IG: n = 18 CG: n = 18 11 years 53% female	Duration: 8 weeks Frequency: 2 times/day 7 days/week Intensity: 30% MIP Volume: 15 min Type: Inspiratory muscle training with a device Supervised: no	Habitual physiotherapy program	MIP ↑ MEP ↔ FEV1 ↔ Forced vital capacity ↔ 6MWT ↔
**Bieli et al. 2017** [29]	IG: n = 11 CG: n = 11 14 years 55% female	Duration: 8 weeks Frequency: 2 times/day 5 days/week Intensity: 60% maximal vital capacity Volume: 10 min Type: Respiratory muscle training with a device Supervised: no	Standard chest physiotherapy	Respiratory muscle endurance test ↑ FEV1 ↔ Forced vital capacity ↔
**Gupta et al. 2019** [30]	IG: n = 25 CG: n = 27 12.5 years 60% male	Duration: 12 months Frequency: 3 days/week Intensity: Unknown Volume: Unknown Type: Home based exercise program of resistance training and plyometric exercises (squats, lunges, push-ups, and jumps) Supervised: no	Routine physical activity	FEV1 ↔ Forced vital capacity ↔ VO_2peak_ ↑

Abbreviations: IG, intervention group; CG, control group; MEP, mouth expiratory pressure; MIP, mouth inspiratory pressure; FEV1, forced expiratory volume 1 s; 6MWT, 6-min-walking test; HR, heart rate; 1RM, one rep maximum. ↔ not statistically significant changes; ↑ statistically significant improvement.

**Table 2:** Outcomes from physical exercise interventions on adults with cystic fibrosis.

Authors, Year	Population	Intervention	Comparison	Outcomes
**Beaudoin et al.** **2017** [31]	IG: n = 8 CG: n = 6 22–57 years 55% female	Duration: 12 weeks Frequency: 3 days/week Intensity: 60–80% VO_2peak_; 30–50% 1RM Volume: 20–40 min aerobic training + 2–3 sets of 8–15 reps of 5–7 exercises for large muscle groups Type: Aerobic and resistance trainingSupervised: no	Routine physical activity	Leg Press ↑ Bench Press ↑ Handgrip strength ↔ Push-ups ↑ Sit ups ↔ Biceps curl ↔
**Reuveny et al.** **2020** [32]	IG: n = 6 CG: n = 5 28 years 55% female	Duration: 8 weeks Frequency: 2 days/week Intensity: 35–70% VO_2peak_ Volume: 45 min Type: HIIT with O_2_ supplementation with cycloergometer Supervised: yes	HIIT without O_2_ supplementation	VO_2peak_ ↔
**Sawyer et al.** **2020** [33]	IG: n = 7 CG: n = 7 31 years 55% female	Duration: 8 weeks Frequency: 3 days/week Intensity: 60–80% Work rate max Volume: 10 min Type: HIIT in cycloergometer Supervised: yes	Routine physical activity	VO_2peak_ ↔ Endurance exercise capacity ↑
**Kaltsakas et al.** **2021** [34]	IG: n = 12 CG: n = 12 32 years 55% male	Duration: 12 weeks Frequency: 3 days/week Intensity: 70% Work rate peak Volume: 30 min Type: Interval exercise training Supervised: yes	Constant load endurance training	MIP ↑ MEP ↑ VO_2peak_ ↑ 6MWT ↑ Quadriceps strength ↑
**Hebestreit et al.** **2022** [35]	IG: n = 60 CG: n = 57 23.5 years 55% female	Duration: 6 months Frequency: 3 days/week Intensity: Vigorous Volume: 60 min Type: Aerobic and resistance training Supervised: no	Routine physical activity	FEV1 ↓ VO_2peak_ ↑ Endurance exercise capacity ↑

Abbreviations: IG, intervention group; CG, control group; 1RM, one rep maximum; HIIT, high-intensity interval training; MEP, mouth expiratory pressure; MIP, mouth inspiratory pressure; 6MWT, 6-min walking test; FEV1, forced expiratory volume 1 s. ↔ not statistically significant changes; ↑ statistically significant improvement; ↓ statistically significant reduction.

## Data Availability

The datasets used and/or analyzed during the current study are avail-able from the corresponding author on reasonable request.
